# Efficacy of azacitidine and trametinib against leptomeningeal melanosis associated with congenital melanocytic nevus syndrome

**DOI:** 10.1186/s40348-026-00233-4

**Published:** 2026-04-13

**Authors:** Lina Raljević, Katalin Komlosi, Ursula Feige, Matthias Eckenweiler, Silke Lassmann, Haroun Bel Hadj Jrad, Martin Werner, Judith Fischer, Cristina Has, Tobias Feuchtinger, Markus Uhl, Charlotte M. Niemeyer, Simone Hettmer

**Affiliations:** 1 Division of Pediatric Hematology and Oncology, Department of Pediatric and Adolescent Medicine, University Medical Center Freiburg, University of Freiburg, Freiburg, Germany; 2 Institute of Human Genetics, University Medical Center Freiburg, University of Freiburg, Freiburg, Germany; 3 Freiburg Center for Rare Diseases, University Medical Center Freiburg, University of Freiburg, Freiburg, Germany; 4 Department of Neuroradiology, University Medical Center Freiburg, University of Freiburg, Freiburg, Germany; 5 Division of Neuropediatrics and Muscular Diseases, Department of Pediatric and Adolescent Medicine, University Medical Center Freiburg, University of Freiburg, Freiburg, Germany; 6 Institute of Clinical Pathology, University Medical Center Freiburg, University of Freiburg, Freiburg, Germany; 7 Department of Radiology, Pediatric Radiology, and Interventional Radiology, St. Josefskrankenhaus, Sautierstraße 1, Freiburg, 79104 Germany; 8 Department of Dermatology and Venerology, University Medical Center Freiburg, University of Freiburg, Freiburg, Germany; 9 Comprehensive Cancer Center Freiburg (CCCF), Medical Center - University of Freiburg, Freiburg, Germany; 10 Pediatrics 1, Martin Luther University Halle, Halle (Saale), Germany

## Abstract

**Background:**

Congenital melanocytic nevus syndrome is a disorder characterized by postzygotic, mosaic NRAS Proto-Oncogene, GTPase mutations. Clinical manifestations include melanotic skin lesions and, optionally, central nervous system melanosis typically noted during early infancy. Affected individuals have an increased risk of developing malignant melanomas at an early age.

**Case:**

We report a child with neurocutaneous melanosis due to this syndrome, who had innumerable nevi at birth and diffuse leptomeningeal thickening. He developed increased intracranial pressure at 4 weeks of age. The nucleoside analogue azacitidine and the Mitogen-Activated Protein Kinase, Kinase inhibitor trametinib were started at 6 weeks of age resulting in rapid reduction of leptomeningeal thickening. At 53 months of age, the patient still takes trametinib and has met all developmental milestones. There has been no evidence of melanoma, and he exhibits minimal residual leptomeningeal changes.

**Conclusion:**

To our best knowledge, this is the first child with this syndrome who has undergone successful therapy to reduce leptomeningeal thickening.

## Introduction

Congenital melanocytic nevus syndrome (CMNS) is a mosaic RASopathy caused by postzygotic *NRAS* variants in codon 61 in up to 80% of all cases [1]. These variants are thought to arise in ectodermal precursor cells, typically leading to melanocyte deposits in the skin and central nervous system (CNS). Patients with CMNS usually present with multiple large or giant cutaneous nevi at birth [1, 2], which may be accompanied by melanocytic lesions in brain parenchyma and/or meningeal membranes in approximately 20–30% of patients [3–5]. For example, neurocutaneous melanosis was reported in 16 of 66 (24,2%) patients with large or giant congenital melanocytic nevi in a published prospective cohort; *NRAS* variants were detected in 75% of patients in this cohort [3]. CNS disease is typically associated with developmental delay, seizures and/or hydrocephalus [2]. There is a high risk that melanocytic lesions in the CNS – especially those involving the leptomeningeal membranes – transform into malignant melanomas at a very young age. These melanomas are considered universally fatal [6]. The risk of malignant transformation depends on the extent of CNS involvement and whether patients are symptomatic. Approximately 50–60% of patients with neurological symptoms develop malignant melanoma [7, 8]; the risk appears to be much lower in asymptomatic patients [9]. Furthermore, most CNS melanomas arise in children with complex neurocutaneous melanosis, i.e. leptomeningeal involvement and/or associated structural abnormalities. Approximately 20% of patients in this group die from primary CNS melanoma. Parenchymatous melanocyte deposits have not been linked to malignancy or death [4]. The child reported here was diagnosed with CMNS with diffuse leptomeningeal melanosis and developed massively raised intracranial pressure at 4 weeks of age. He had an exceptional response to experimental combination therapy consisting of azacitidine and trametinib. At the age of 53 months he is alive and well with minimal residual leptomeningeal thickening.

## Case report

**Fig. 1:**
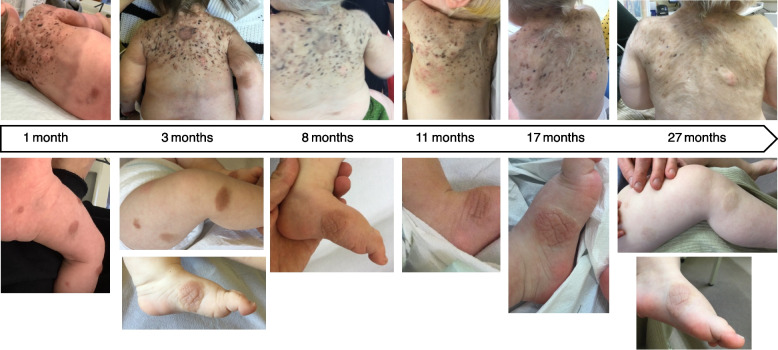
Skin findings. A giant congenital melanocytic nevus on the upper back and multiple smaller satellite nevi on the head, arms, and legs were present at birth. Follow-up pictures at 1 month, 3 months and 8 months, 11 months, 17 months and 27 month of age show that the color of most of the skin lesions faded, while excessive hair grew over time

A male infant was born at term to healthy, non-consanguineous Caucasian parents. At birth, the boy had giant, light brown melanocytic nevi with multiple irregular dark brown and nodular areas on the neck, upper thorax, and arms, as well as numerous satellite nevi on the head, arms, and legs (Fig. 1). Melanocytic lesions covered approximately 20% of his body surface. Family history was notable for malignant melanoma diagnosed in the maternal grandfather at age 40. The patient’s 4-year-old brother was healthy and had no abnormal skin findings. Screening magnetic resonance imaging (MRI) of the neuroaxis was performed at the age of 4 weeks. The MRI revealed diffuse, contrast medium-absorbing lesions compatible with leptomeningeal melanosis. Frequent episodes of oxygen desaturation were observed during monitoring and interpreted as apneic episodes due to massively raised increased intracranial pressure. A ventriculoperitoneal shunt was placed. Repetitive sampling and cytology examinations of cerebrospinal fluid (CSF) did not reveal any malignant cells, but neurological symptoms and extensive CNS melanosis—including widespread leptomeningeal lesions—indicated > 50% melanoma risk.

Next generation sequencing (NGS, TruSight Oncology 500; Illumina) of DNA obtained from a biopsy of a dermo-subcutaneous melanocytic lesion revealed the classic pathogenic (hotspot) p.Gln61Lys variant in exon 3 of the *NRAS* gene (NM_002524.4: c.181C > A, NP_002515.1; p.Gln61Lys). The allele frequency (AF) of this known activating variant was 35%. Further genetic analysis using germline DNA derived from a peripheral blood sample did not identify the *NRAS* variant, indicating its postzygotic origin. Yet, a heterozygous variant, p.Glu318Lys, in the *MITF1* (Microphthalmia-Associated Transcription Factor 1) gene was identified in both the skin and blood samples (NM_000248.3: c.952G > A, p.Glu318Lys, AF of 50%). The likely pathogenic *MITF1* variant represented a heterozygous germline mutation inherited through the maternal line.

**Fig. 2:**
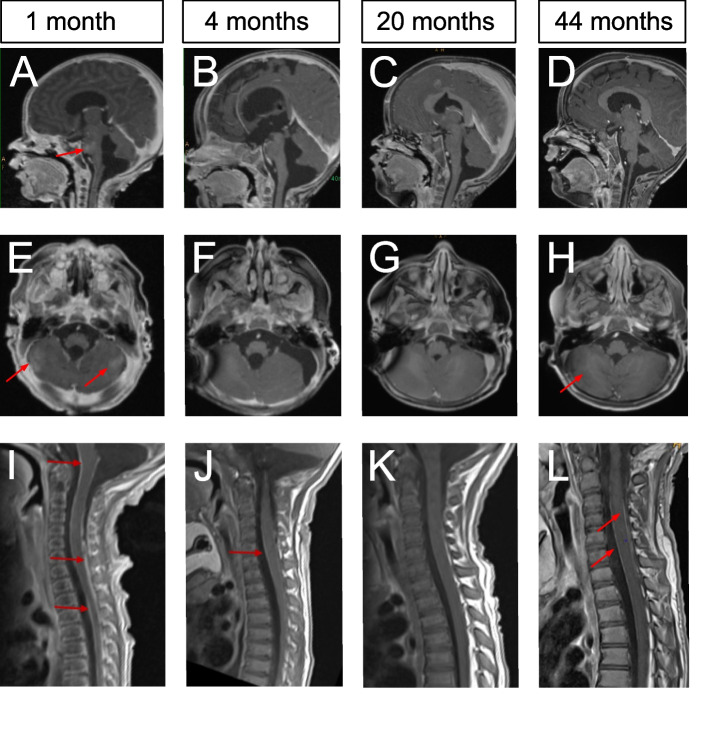
Leptomeningeal thickening and parenchymatous lesions, presumed to represent melanosis. MRI imaging of the brain and spine obtained at the ages of one month (**A**, **E**, **I**), 4 months (**B**, **F**, **J**), 20 months (**C**, **G**, **K**) and 44 months (**D**, **H**, **L**). **A**-**D** Post-contrast, fat-saturated 3D T1-weighted MPRAGE sequences, acquired in the sagittal plane with (**E**–**H**) multiplanar reformating into the axial view demonstrate (**A**, **E**) extensive contrast-enhancing lesions involving the right pontine and cerebellar regions (marked by red arrows) at the age of one month associated with cerebrospinal fluid (CSF) accumulation and hydrocephalus, as well as a space-occupying cystic lesion in the posterior cranial fossa, ventral to the lower margin of the fourth ventricle. Melanin in tumor cells may cause shortened T1 relaxation times and act as an endogenous contrast agent. Melanin-expressing lesions regularly are expected to be signal-intense on T1-weighted MRI-images, but this phenomenon is not observed here. (**B**, **F**) Rapid, near-complete regression of the parenchymatous lesions in the right pons and cerebellum was noted at 4 months of age. The hydrocephalus was more prominent. (**I**-**L)** T1-weighted turbo spin echo (TSE) sequences of the spine, also acquired in the sagittal plane, were obtained following intravenous contrast administration. (**I)** Diffuse intraspinal leptomeningeal enhancement was noted at one month of age. (**J)** There was marked regression of the leptomeningeal enhancement along the spinal cord at 4 months of age; only one minimal residual focus (marked by a red arrow) was visible. MRI images obtained at (**C**, **G**, **K**) 20 months of age (2 months after discontinuation of azacitidine) and (**D**, **H**, **L**) at 44 months of age demonstrate residual long-term changes in the pons and myelon (marked by red arrows). The hydrocephalus has regressed

Individualized drug therapy with azacitidine (75 mg/m^2^ per dose daily by subcutaneous injection for 7 consecutive days in 28-day cycles) and trametinib (0.035 mg/kg per dose daily per os) as previously described [10] was initiated on day of life 47 and followed by rapid reduction of the extensive leptomeningeal thickening (Fig. 2). Treatment with azacitidine and trametinib was well-tolerated except for an intermittent eczematous skin rash, temporarily slow longitudinal growth and intermittent nausea/vomiting. After 18 cycles of azacitidine and in the setting of minimal, radiologically stable leptomeningeal thickening, azacitidine treatment was discontinued to limit discomfort from subcutaneous injections and nausea/vomiting temporally associated with azacitidine. The patient continued oral trametinib treatment. At the age of 45 months (26 months after discontinuing azacitidine), MRI of the neuroaxis continued to demonstrate minimal, residual leptomeningeal changes (Fig. 2). The color of most of the skin lesions has paled over time. Many of them exhibit excessive hair growth, and the nodular areas have decreased (Fig. 1). The child is on the 69th percentile for height and on the 89th percentile for weight. He has met all developmental milestones. Regular cardiology and ophthalmology examinations have yielded age-appropriate results. No endpoint for the treatment with trametinib has been established at this time.

The patient’s hydrocephalus is likely due to impaired CSF resorption secondary to his underlying condition. Shunt complications included an episode of streptococcus pneumoniae meningitis and multiple episodes of shunt dysfunctions (most recently at 52 months of age). Shunt dysfunction was associated with symptoms of increased intracranial pressure with bilateral papilledema, which resolved following shunt revision. It is anticipated that the patient will require a ventriculoperitoneal shunt permanently.

## Discussion

In summary, we report an exceptional response to experimental treatment with azacitidine and trametinib in a male infant with CMNS and widespread, symptomatic leptomeningeal melanosis. To the best of our knowledge, this is the first child with CMNS, who has undergone successful therapy to reduce leptomeningeal thickening.

Our group previously reported the case of a 32-month-old boy with CMNS and advanced CNS melanoma, who had an observable clinical and radiological response to treatment with azacitidine and trametinib [10]. Although he succumbed to rapidly progressive disease after 5 months of therapy, response to the trametinib and azacitidine combination appeared to be of longer duration than that published for trametinib alone [11, 12]. In vitro studies using *NRAS*-mutated melanoma cells suggested that combined exposure to trametinib and azacitidine postponed the development of trametinib resistance in *NRAS*-mutated melanoma cells; the trametinib and azacitidine combination had synergistic effects against trametinib-resistant cells [10]. The decision to treat the patient reported here was based on these observations.

Typical skin and CNS findings led to the diagnosis of CMNS in the patient reported here. This was supported by the detection of a classical activating p.Gln61Lys *NRAS* variant in DNA obtained from a melanocytic skin nevus but not from blood. Biopsies from the patient’s leptomeningeal disease manifestations were never obtained, which represents a limitation of this case. We suspect that CNS melanoma was not present at the time of diagnosis, and there is no histological evidence that the patient developed melanoma during treatment and follow-up as of today. It is possible that the patient would have remained melanoma-free without therapy. Yet, the risk of developing CNS melanoma in a severely affected, symptomatic infant with widespread leptomeningeal melanosis was estimated to be at least 50% and likely much higher in the long run. Treatment with azacitidine and trametinib was started to reduce CNS melanosis, allow for normal development, and prevent malignant transformation. The patient’s long-term prognosis and risk of sequelae from azacitidine and trametinib exposures at such a young age remain unclear.

Combination therapy with azacitidine and trametinib was well-tolerated except for an intermittent eczematous skin rash (thought to be secondary to trametinib), temporarily reduced longitudinal growth (possibly related to frequent shunt complications and streptococcus pneumoniae meningitis during this time) and intermittent nausea/vomiting. Residual leptomeningeal changes were radiologically stable over time, and nausea/vomiting worsened over time and appeared to be temporally associated with subcutaneous azacitidine administration. Thus, azacitidine treatment was discontinued after 18 cycles to reduce discomfort related to subcutaneous injections and drug-induced nausea. The molecular events resulting in melanoma development may be linked to a developmentally susceptible age window, but it is important to note that melanoma risk in CMNS persists throughout life [13]. The patient continues to take trametinib, and no endpoint has been set for the treatment with trametinib yet.

The patient inherited the heterozygous *MITF1* germline variant NM_000248.3: c.952G > A, p.Glu318Lys through the maternal line. This variant is the most common known pathogenic *MITF* variant and has been found in individuals with cutaneous melanoma and renal cell carcinoma [14]. Heterozygous pathogenic *MITF1* germline variants have been described in familial melanoma [14–16], and hypermethylation of *MITF1* plays an important role in melanoma development [17]. Yet, the role of *MITF1* germline variants in children with neurocutaneous melanosis is unclear. It might represent an additional risk factor for melanoma development in our case, since melanoma occurred in other members of the maternal family.

Melanoma in children and adolescents is rare and comprises a heterogeneous group of melanocytic neoplasms. The molecular landscape of conventional melanomas in children mimics the UV-induced mutational spectrum found in melanomas in adults, including driver mutation in the RAS/RAF/mitogen-activated protein kinase pathway, alterations in the phosphatase and tensin homolog (*PTEN*) tumor suppressor gene and, notably, transcription- activating mutations of the telomerase reverse transcriptase (*TERT*) promoter (*TERT-p*) resulting in increased TERT expression [18]. By contrast, melanomas arising in association with large congenital nevi lack *PTEN* and *TERT-p* gene alterations [18]. Instead, CpG sites in the *TERT-p* region of CMNS melanomas are highly methylated, consistent with an alternative mechanism of TERT reactivation [19]. Indeed, *TERT-p* CpG hypermethylation correlates with high TERT expression in CMNS melanomas.

It is important to note that, in a published study [19], *TERT-p* CpG hypermethylation was detected in CMNS melanomas, but not in the nodules, which form within giant congenital nevi of the skin and typically consist of clonal proliferations of cells. Thus, we hypothesize that congenital melanocytic nevi (CMN) versus clonal proliferations of nevus-associated cells versus CMN-associated melanomas represent a continuous disease spectrum, with melanomas developing from nevi through clonal evolution. Reduction and/or prevention of *TERT-p* CpG hypermethylation by exposure to the methyltransferase inhibitor azacitidine may explain the efficacy of the drug – administered in combination with trametinib – in the patient reported here.

## Data Availability

The datasets relevant to this report are not publicly available, to protect the patient’s privacy, but are available from the corresponding author upon reasonable request in an anonymised form.

## Abbreviations

AF: Allele frequency
CMN: Congenital melanocytic nevus
CMNS: Congenital melanocytic nevus syndrome
CNS: Central nervous system
CpG: Cytosine (C)—phosphate (p)—Guanine (G)
CSF: Cerebrospinal fluid
DNA: Deoxyribonucleic acid
MEK: MAPK/ERK kinase (MAPK = Mitogen-Activated Protein Kinase)
*MITF1*: Microphthalmia-Associated Transcription Factor 1
MPRAGE: Magnetization-Prepared Rapid Gradient-Echo
MRI: Magnetic resonance imaging
NGS: Next generation sequencing
*NRAS*: Neuroblastoma RAS proto-oncogene
*PTEN*: Phosphatase and tensin homolog
RAF: RAF (Rapidly Accelerated Fibrosarcoma) serine/threonine kinases
*RAS*: RAS (RAt Sarcoma) proto-oncogene
*TERT*: Telomerase reverse transcriptase
*TERT-p*: Telomerase reverse transcriptase promoter
TSE: Turbo spin echo
UV: Ultraviolet
